# 

**Published:** 1894-02

**Authors:** 


### CONTENTS:

ORIGINAL ARTICLES: PAGE

On the Anatomy of Tænia Crassicollis Rud. By A. E. Loveland, B.A. .......... 67
Clinical Diagnosis in Veterinary Ophthalmology By L. Van Es, V.S. .......... 90
Unsuspected Poisoning by Sterilized Meat and Milk of Tuberculous Animals. By James Law, F.R.C.V.S. .......... 101
Professional Etiquette. By Claude D. Morris, V.S. .......... 109
Hæmoglobinuria. Extract from Letter of John M. Eshlemau, V.M.D. .......... 115
Pyo-septhamie in Foals Caused by Phlebitis Umbilicalis. By John Wende,,V.S. .......... 116
Nails in Feet. By John A. Bell, V.S. .......... 119
Choking and the Advantages of Belladonna in Treatment of Same. By John Meikle, M.R.C.V.S. .......... 121
A Complicated Wound of the Leg. By John M. Eshleman, V.M.D. .......... 122
Obituary.—Dr. Robert C. Hutchings .......... 123

EDITORIAL:

Current Literature for Veterinarians—Continued .......... 124
New York Veterinary Societies .......... 127
Pennsylvania State Veterinary Medical Association .......... 127

SOCIETY PROCEEDINGS:

Massachusetts Veterinary Association .......... 128
German Veterinary Medical Association from New York and Vicinity .......... 134
Keystone Veterinary Medical Association .......... 134
New York State Veterinary Medical Society—Fourth Annual Meeting .......... 136
United States Veterinary Medical Association .......... 154
Veterinary Medical Association of New York County .......... 155
Second Annual Meeting of the North Dakota Medical Association .......... 157
Ontario Veterinary Medical Society .......... 159

